# Evaluation of root-knot nematode resistance assays for sugarcane accession lines in Australia

**DOI:** 10.21307/jofnem-2021-006

**Published:** 2021-03-01

**Authors:** S. A. Bhuiyan, K. Garlick

**Affiliations:** Sugar Research Australia, 90 Old Cove Road, Woodford, Qld, 5414, Australia

**Keywords:** Root-knot nematode, *Meloidogyne javanica*, Resistance, Sugarcane, Disease screening

## Abstract

Root-knot nematode (*Meloidogyne javanica*) is an important pathogen of sugarcane and can cause significant yield loss in sandy soil. Resistance to root-knot nematode is not common in commercial cultivars of sugarcane. In order to find new sources of resistance for root-knot nematodes, Sugar Research Australia (SRA) has screened new sets of accession lines derived from introgression breeding between wild relatives of sugarcane and commercial parents, and sugarcane accession lines from advanced stages of the core selection programs. This study aimed to determine the most suitable assessment parameter for comparing resistance of accession lines and cultivars. Eight screening trials were conducted from 2012 to 2019. Three parameters were measured on individual plants grown in pots inoculated with an initial population of *M. javanica* eggs, (i) number of eggs per plant, (ii) number of eggs per g of roots, and (iii) visual ratings of galls on roots. Resistance parameter, eggs per plant was repeatable among trials and had less variations within a trial than the other parameters. Eggs per g of roots was negatively correlated (*r* = −0.19 to −0.74, *p* < 0.05) with root biomass in seven out of eight trials, and with shoot biomass in four out of seven trials (*r* = −0.24 to −0.47, *p* < 0.001). Visual rating of galls showed inconsistent (positive or negative) correlations with shoot and root biomass. No significant correlations were found between number of eggs per plant and shoot or root biomass of test accession lines. Based on repeatability and variability of resistance parameters, eggs per plant was the most suitable parameter to compare and select sugarcane accession lines for resistance to *M. javanica*.

Plant-parasitic nematodes are major constraints to sugarcane production worldwide (Ramouthar and Bhuiyan, 2018). In Australia, plant-parasitic nematodes cause 5 to 20% yield loss per year, costing over $80 million in productivity (Blair and Stirling, 2007). The most important nematodes of sugarcane in Australia are root-lesion nematode (*Pratylenchus zeae*) and root-knot nematode (*Meloidogyne javanica*). *Meloidogyne javanica* is primarily abundant in sandy soil and can cause significant yield loss ((Blair and Stirling, 2007; Blair et al., 1999a, b).

Rotation crops or fallowing provide short-term protection, but *M. javanica* populations increase to damaging levels within 12 months when sugarcane is replanted (Stirling, 2008; Stirling et al., 2001). Also, many rotation crops such as tomatoes, soybean, and cucurbits are hosts of *M. javanica* (Trudgill and Blok, 2011). Nematicides are relatively expensive and only reduce nematode populations for a few months in sugarcane (Blair and Stirling, 2007). A preliminary study found no commercial varieties of sugarcane were resistant to root-knot nematodes in Australia (Stirling et al., 2011).

Modern sugarcane varieties were derived from crosses between noble cane *Saccharum officinarum*, and a wild relative of sugarcane *S. spontaneum*, which were then backcrossed to *S. officinarum* or other complex hybrids (Cox et al., 2000). These interspecific hybridisations provided resistance to diseases and tolerance to a range of biotic and abiotic stresses (Cox et al., 2000). Those handful of early hybrid sugarcane lines formed the basis of sugarcane breeding programs around the world (Arceneaux, 1967; Roach, 1989), and it has been suggested that their repeated use for developing new cultivars of sugarcane has led to deteriorating genetic gain in terms of yield, pest, and disease resistance (Kandel et al., 2018).

A collaborative research project between Australia and China in the late 1900s and early 2000s used new sources of wild relatives of sugarcane, *Erianthus* spp. and *S. spontaneum* lines to generate new introgression families (Foreman et al., 2007). In early 2003, the progenies of the introgression accessions were imported to Australia for further study and crossings. Unfortunately, the parent materials from China could not be imported because of the quarantine restriction. A preliminary study indicated that those lines were resistant to a range of root pathogens such as pachymetra root rot (Magarey and Croft, 1996), root-knot, and root-lesion nematodes (Stirling et al., 2011) and were tolerant to water logging and drought (Foreman et al., 2007). A research project was commenced by Sugar Research Australia (SRA, formerly BSES) in 2011 to determine the level of nematode resistance in germplasm from domesticated and wild species and in the progenies derived from crosses between sugarcane and both *Erianthus* spp. and *S. spontaneum* lines with commercial sugarcane. The first component of that work was to develop methods for screening large numbers of sugarcane lines against plant-parasitic nematodes. Initial research by the authors determined trial conditions, suitable potting media, and extraction methods for root-knot nematodes (Bhuiyan et al., 2014). The other important aspect was to formulate an assessment method for nematode resistance that is reliable and suitable for screening large numbers of progeny lines.

The parameter(s) used to screen progeny for disease resistance needs to be realistic, and repeatable. The reproductive capabilities of nematodes on sugarcane accession lines were used to measure the resistance to nematodes in screening trials. In this process, each test line was inoculated with 5,000 eggs of *M. javanica* and the final population (eggs) retrieved from the inoculated plants after 12 weeks was determined (Bhuiyan et al., 2016; Stirling et al., 2011). *Meloidogyne javanica* is an obligate parasite of plants and their ability to multiply may be limited by the availability of roots. Measuring shoot and root growth can be used to assist in comparisons between accession lines (Stirling et al., 2011). If accession lines with similar root mass have different numbers of eggs, then the difference is most likely due to resistance. However, if an accession line has a smaller root system, the lower number of eggs could be due to the limited root mass available to the nematodes as a food source.

Scoring the visual symptoms of nematode infection (e.g., galling) has been used by some researchers to rapidly rate plants for resistance to nematodes, particularly when the symptoms are obvious. Wang and Goldman (1996) used a 0 to 5 scale to rate the severity of galling on carrots inoculated with *M. hapla*. Shepherd (1979) used a root-knot index to score cotton plants for the severity of symptoms caused by *M. incognita*. In some crops it has been useful to obtain quantitative data on egg number per g of roots and this measure has given a better indication of root-knot nematode resistance than either gall or egg mass numbers (Luzzi et al., 1987). In sugarcane screening experiments, visual rating of galling showed good correlations with eggs per plants for root-knot nematode (Bhuiyan et al., 2016).

This study examined some of the parameters used to evaluate root-knot resistance in sugarcane accession lines from 2012 to 2019 in Australia to determine suitable parameters that provide reliable and repeatable results.

## Materials and methods

### Trial information

A total of 802 sugarcane accession lines were tested against *M. javanica* from 2012 to 2019 in eight trials at SRA Woodford Pathology Farm, Woodford, Queensland (26.9550° S, 152.7780° E) (Table 1). Among these, 648 lines (year 2012-2016) were from introgression populations, and 154 lines (year 2017-2019) were from the core breeding germplasm populations of SRA. In total, 22 accession lines, mainly commercial varieties/parents (including two susceptible controls), were incorporated in multiple trials to obtain data on their resistance to *M. javanica* (Table 2).

**Table 1.** Trial names, trial year, number of accession lines and types of sugarcane populations screened for root-knot nematode (*Meloidogyne javanica*) resistance from 2012 to 2019.

Trial name	Trial year	Number of accession lines	Source
Nem19-1	2019	42	Core breeding program
Nem18-1	2018	56	Core breeding program
Nem17-1	2017	56	Core breeding program
Nem16-2	2016	95	Introgression population
Nem15-1	2015	112	Introgression population
Nem13-1	2013	138	Introgression population
Nem13-2	2013	155	Introgression population
Nem12-1	2012	148	Introgression population
Total accession lines	–	802	–

**Table 2.** List of 22 sugarcane accession lines used in multiple trials with root biomass, and nematode resistance parameters.

Accession	Type	No. of trials^a^	Root biomass (g)	Relative eggs per plante (%)	Relative eggs per g root (%)	Visual gall rating
IJ76-333	*Erianthus* sp^b^	5	25.3±12.5d	12.4±2.19	25.6±18.99	1.3±0.06
IJ76-370	*Erianthus* sp	5	23.2±11.16	10.7±1.19	7.8±3.26	1.1±0.1
KQ228	Core	8	21.0±7.66	43.2±6.62	23.6±9.1	2.6±0.17
Q135c	Core	7	12.9±4.78	125.2±27.71	145.0±67.32	3.2±0.2
Q138	Core	7	15.2±4.61	40.0±8.68	25.5±10.19	2.7±0.35
Q183	Core	4	6.8±4.14	67.8±11.13	74.9±37.44	3.0±0.19
Q200	Core	6	10.9±5.07	144.2±43.27	115.7±40.94	2.9±0.2
Q208c	Core	8	16.2±5.02	77.9±8.56	60.6±28.92	3.0±0.23
Q231	Core	4	17.6±12.07	36.4±14.26	16.6±10.16	3.0±0.51
Q232	Core	6	14.3±5.13	55.5±14.06	43.8±21.41	2.7±0.31
Q238	Core	4	16.2±7.96	53.5±14.84	25.8±12.79	3.4±0.18
Q240	Core	5	20.4±6.38	101.4±25.49	29.0±13.63	2.8±0.3
Q241	Core	4	8.0±6.59	30.8±8.73	88.1±49.45	3.1±0.43
Q242	Core	4	11.8±6.31	64.6±28.2	30.5±14.41	3.0±0.57
Q245	Core	6	9.4±4.78	45.7±7.38	58.9±28.38	2.7±0.25
Q248	Core	4	16.2±8.99	139.2±35.96	51.5±24.73	3.3±0.28
Q249	Core	4	13.1±7.65	24.6±3.16	17.0±10.41	3.0±0.34
Q251	Core	4	12.6±4.82	90.1±21.37	30.7±11.44	2.7±0.37
QBYN05-20563	Introgression	4	24.1±3.03	11.7±3.4	3.7±1.83	2.6±0.22
SRA1	Core	4	6.2±3.65	43.2±11.22	81.7±57.66	2.7±0.24
SRA2	Core	4	3.5±1.66	43.9±12.05	79.9±46.09	2.4±0.14
SRA3	Core	4	8.5±5.13	60.7±14.42	89.0±63.14	2.9±0.4

**Notes:** ^a^Number of trials the accession was included; ^b^wild relative of sugarcane; ^c^susceptible controls; ^d^Mean±SE; ^e^relative eggs/plant or g root = *T*/*S*×100, where *T* is mean eggs per plant or g root for test accession line, and *S* is mean eggs per plant or g root for two susceptible controls (Q208 and Q135).

**Figure 1:**
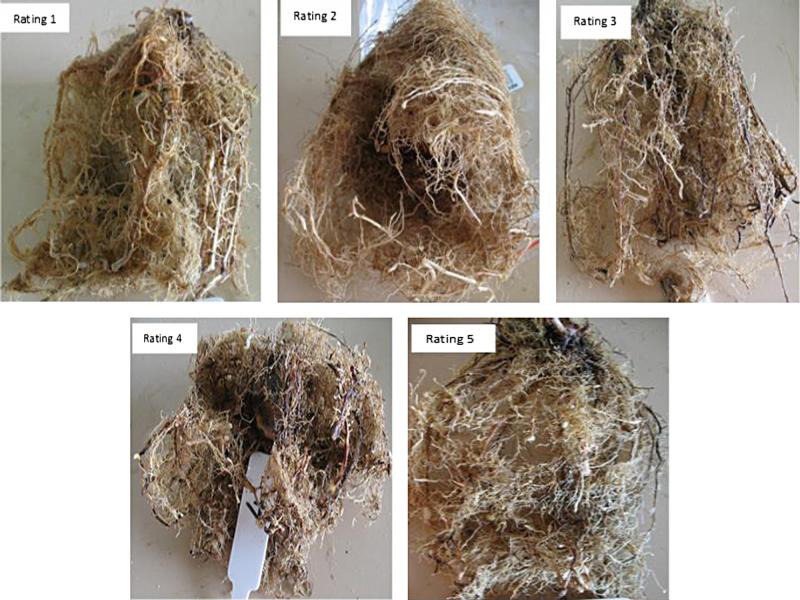
Examples of root-knot nematode gall ratings for sugarcane based on percentage of root system with galls. 1 = ≤1 to 2%, 2 = 2 to 25%, 3 = 25 to 50%, 4 = 51 to 75%, 5 ≥ 75% (modified from Shepherd 1979).

Details of trial procedures and design of experiments were described elsewhere (Bhuiyan et al., 2014, 2016; Croft et al., 2015). In short, all accession lines were collected from the SRA germplasm collection, Meringa, Queensland. Stalks of accession lines were stripped of leaves and leaf sheaths, cut into one-budded setts using an electric saw, hot water treated at 50^o^C for 30 min to eliminate systemic fungal and bacterial diseases and placed in a germination chamber in trays with moist vermiculite. Germinated young plants were planted to 700 ml pots filled with sterilized fine white washed sands (Caboolture Gravel & Landscapes, Morayfield, Qld), maintained in a glasshouse for inoculation. Each trial was set out on air conditioned benches using a randomized complete block design in five replicate pots, and one plant per replication. Two data loggers (Thermodata Pty Ltd, Brisbane, Australia) were included in each trial by burying them approximately 5 cm deep in potting media to record the temperature of the potting media during the trial period. Two commercial varieties Q208 and Q135 that were highly susceptible to *M. javanica* were included in each trial as controls for comparison. The *M. javanica* were reared in the roots of tomato plants in a glasshouse as described by Van den Bergh et al. Eggs of *M. javanica* were extracted from the tomato plants using the bleach method (Stirling et al., 2011). Inoculation was done by applying approximately 5,000 eggs per pot. Eggs were added in 5 ml deionized water at the base of the plant in two holes using a pipette. Plants were maintained for approximately 12 weeks before harvesting and assessing for nematode population and resistance based on number of eggs per plant, number of eggs per g of fresh roots, and visual rating of gall severity. Root and shoot biomass were measured for each plant (Bhuiyan et al., 2016, 2019). For visual ratings, the roots were washed free of sands and visually rated for galling using a previously described method (Shepherd, 1979) with slight modification on a 0 to 5 scale based on the percentage of roots with galls, as shown in Figure 1, where 0 = no galls, 1 = ≤1 to 2%, 2 = 2 to 25%, 3 = 25 to 50%, 4 = 51 to 75%, 5 ≥ 75%.

For the extraction of eggs, roots were washed free of sands, submerged in 1% NaOCl (bleach) and agitated for 5 min. The bleach solution was then poured through two sieves (a 150 μm sieve over a 38 μm sieve), with eggs being collected on the lower sieve, and finally washed into a 30-ml plastic vial. Extracted egg samples were stored at 6°C until counting. Enumeration of a subsample of nematode eggs were performed under a compound microscope (10×- 40×) using a Hawksley slide counting chamber of 1 ml capacity.

### Data analysis

The number of eggs per plant was recorded after the enumeration, and the number of eggs per g of root was estimated from the total number of eggs present in the pot divided by the fresh weight of the root system. Data were analyzed by fitting a linear mixed model to all datasets using *Proc Mixed* in SAS version 9.4 (SAS Institute, Cary, NC). For each trial, accession lines were treated as a fixed effect. Block (replication) and the error term (residual) were treated as a random effect. The interactions of the fixed effects were included in the model. Degrees of freedom were adjusted using the Kenward–Roger method (Kenward and Roger, 1997) and normality of residuals was tested using *Proc Univariate* of SAS. The number of eggs per plant, and the number of eggs per g root data were log-transformed (ln (*x*+1)) before analysis. *Proc Corr* of SAS was used to calculate correlations between mean of nematode resistance parameters and root or shoot biomass. *Corr plot = matrix* in SAS was used to calculate the correlation matrix to determine the linear relationship of root and shoot biomass with nematode parameters. On the basis of the linearity, *Proc Reg* of SAS was used to determine the relationship between root biomass and eggs per g of roots. The percent of coefficient of variation (CV) within each trial for root and shoot biomass, and visual ratings for gall were calculated as CV = 100%(*σ*/*µ*), where *σ* and *µ* are the standard deviation and mean, respectively. As for the log-transformed data a formula:

$$\mathrm{CV}=100\%\sqrt{e^{\ln\;{\left(10\right)}^{2\;}\sigma^{2}}-1},$$

where *σ* is the standard deviation of the log-transformed data was used (Canchola et al., 2017). In total, 22 accession lines, as described earlier, were selected to determine the repeatability among the trials in relation to nematode resistance parameters (Table 2). Each accession line was included in at least four of the screening trials. SAS *Corr* in *Fisher* command was used to transform the data to normalize the distribution and stabilize the variance (Steel and Torrie, 1960).

## Results

### Repeatability and reliability

The CVs for sugarcane biomass and nematode parameters varied among trials. The highest variations were in root biomass, ranging from 30 to 49% (Table 3). The CVs in the nematode parameters, eggs/plant, and eggs/g roots were higher in the early trials in years 2012 (Nem12-1) and 2013 (Nem13-1 and Nem13-2), ranging from 25 to 47%. In all other trials, the nematode parameter, eggs/plant had the lowest variations ranged from 2.3 to 9.2%, followed by eggs/g root (3.2-12%).

**Table 3.** Coefficient of variance (%) among shoot biomass, root biomass, visual ratings, number of eggs per plant and number of eggs per g of roots in each trial.

Trial name	Shoot biomass	Root biomass	Visual rating	Eggs per plant	Eggs per g root
Nem19-1	21.5	36.3	20.5	2.3	6.4
Nem18-1	20.7	38.7	19.3	2.8	3.2
Nem17-1	27.3	48.7	20.5	2.7	9.9
Nem16-2	-	38.2	22.1	6.0	8.7
Nem15-1	20.9	38.3	30.0	9.2	11.9
Nem13-1	21.5	29.6	22.4	28.6	47.1
Nem13-2	27.9	37.0	22.6	25.3	27.8
Nem12-1	24.0	37.5	29.3	25.0	25.5

In 22 accessions, eggs per plant were more repeatable than eggs per g of root and visual ratings across the trials (Table 4). In 18 out of 28 possible trial combinations that include the same accessions, correlation of eggs per plant among trials were significant (*p < *0.05). In 14 out of 28 possible trial combinations eggs per g of root for the same accession lines were correlated among the trials (*p < *0.05). Only 11 out of 28 combinations for the same accession lines, was there a correlation between visual gall ratings among trials were significant (*p < *0.05) with *r* values ranged from 0.72 to 0.92. Root biomass was variable in 22 accessions, varied from just over 3 g in commercial variety SRA2 to 25.3 g in IJ76-333 (*Erianthus* sp) (Table 2). Two wild accessions (IJ76-333 and IJ76-370) and only introgression accession QBYN05-20563 were highly resistant, supporting the lowest number of eggs per plant (3.7-12.4%) in relation to susceptible controls. Entries IJ76-370 and QBYN05-20563 supported only 7.8 and 3.7% eggs per g root compared to susceptible control, respectively. All other accessions had close to 20% or more eggs per plant or g root compared to susceptible controls. Among commercial varieties, Q231, Q241, and Q249 had less than 40% relative eggs per plant compared to the controls.

**Table 4.** Pearson correlation coefficients among 22 accession lines to measure the repeatability among trials in relation to nematode (*Meloidogyne javanica*) resistance parameters.

Trial	by Trial	No. of accession lines^a^	Eggs per plant^b^	Eggs per g root	Gall rating
Nem12-1	Nem13-1	6	0.99***	0.89*	0.92**
Nem12-1	Nem13-2	6	0.98***	0.94**	0.86*
Nem12-1	Nem15-1	6	0.10 ns	0.20 ns	0.56 ns
Nem12-1	Nem16-2	7	0.98***	0.76*	0.20 ns
Nem12-1	Nem17-1	6	0.94**	0.95**	0.86*
Nem12-1	Nem18-1	8	0.93**	0.63 ns	0.64 ns
Nem12-1	Nem19-1	8	0.87**	0.92**	0.86**
Nem13-1	Nem13-2	3	0.99 ns	0.82 ns	–
Nem13-1	Nem15-1	12	0.47 ns	0.31 ns	0.27 ns
Nem13-1	Nem16-2	12	0.92***	0.10 ns	0.35 ns
Nem13-1	Nem17-1	10	0.74*	0.60 ns	0.72*
Nem13-1	Nem18-1	8	0.91**	0.71*	0.82*
Nem13-1	Nem19-1	7	0.69 ns	0.59 ns	0.89**
Nem13-2	Nem15-1	5	−0.02 ns	0.33 ns	0.71 ns
Nem13-2	Nem16-2	6	0.95**	0.80 ns	−0.30 ns
Nem13-2	Nem17-1	5	0.92*	0.95*	0.68 ns
Nem13-2	Nem18-1	6	0.90*	0.46 ns	0.68 ns
Nem13-2	Nem19-1	6	0.86*	0.83*	0.87*
Nem15-1	Nem16-2	18	0.19 ns	0.29 ns	−0.30 ns
Nem15-1	Nem17-1	15	0.25 ns	0.12 ns	0.19 ns
Nem15-1	Nem18-1	10	0.51 ns	0.66*	0.16 ns
Nem15-1	Nem19-1	13	0.66*	0.60*	-0.48 ns
Nem16-2	Nem17-1	14	0.74**	0.28 ns	0.06 ns
Nem16-2	Nem18-1	10	0.81 ns	0.63*	−0.38 ns
Nem16-2	Nem19-1	12	0.43 ns	0.40 ns	0.27 ns
Nem17-1	Nem18-1	8	0.97***	0.77*	0.82**
Nem17-1	Nem19-1	13	0.92***	0.86***	0.79**
Nem18-1	Nem19-1	10	0.93***	0.71*	0.88**

**Notes:** ^a^Common accession lines between the two trials; ^b^significant (*p* value) *≤0.05, **≤0.01, ***≤0.0001, ns = no significant.

### Correlations of root or shoot biomass with nematode resistance parameters

There were no significant correlations of nematode eggs per plant with root and shoot biomass in any of the eight trials (Table 5). Nematode eggs per g of root were negatively correlated (*r* = −0.24 to −0.47, *p*≤0.001) with shoot biomass in four out of seven trials. Low (*r*≤−0.19, *p*≤0.05) to strong (*r* = −0.65 and −0.74, *p* < 0.0001) negative correlations of eggs per g of root were observed with root biomass in seven out of eight trials. In two out of seven trials, visual ratings had moderate positive correlations with shoot biomass (*r* = 0.24 and 0.30, *p < *0.05) and the rest of the trials had no significant correlations. Moderate negative correlations were observed between visual rating and root biomass in two out of eight trials, and one trial showed moderate positive correlations between these two parameters.

**Table 5.** Pearson correlation coefficients to compare relationships of nematode (*Meloidogyne javanica*) resistance parameters with sugarcane shoot biomass and root biomass.

	Eggs per plant		Eggs per g root		Visual rating	
Trial name	Shoot biomass	Root biomass	Shoot biomass	Root biomass	Shoot biomass	Root biomass
Nem19-1	−0.08 ns^a^	−0.27 ns	−0.47**	−0.74***	−0.14 ns	−0.41**
Nem18-1	0.01 ns	−0.03 ns	−0.36**	−0.45**	0.01 ns	−0.08 ns
Nem17-1	0.070 ns	−0.26 ns	−0.42**	−0.65***	−0.004 ns	−0.26 ns
Nem16-2	–	0.13 ns	–	−0.44***	–	0.11 ns
Nem15-1	−0.093 ns	−0.07 ns	−0.24**	−0.28**	−0.19 ns	−0.12 ns
Nem13-1	0.15 ns	0.14 ns	0.12 ns	−0.06 ns	0.30**	0.21*
Nem13-2	0.19 ns	0.02 ns	0.08 ns	−0.19*	−0.10 ns	−0.40***
Nem12-1	0.18 ns	0.06 ns	−0.02 ns	−0.23**	0.24*	−0.04 ns

**Note:** ^a^Significant (*p* value) *≤0.05; **≤0.001; ***≤0.0001; ns = no significant.

### Relationships of root biomass with eggs per g of roots

**Figure 2:**
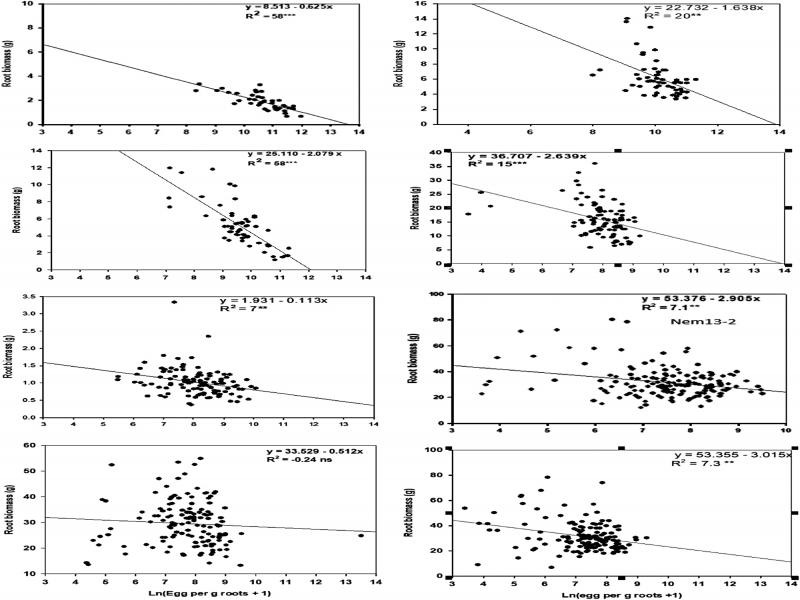
Regressions to show the relationship between root biomass and ln(eggs per g roots+1) in eight nematode trials.

The relationships between eggs per g of root and root biomass varied among the trials (Fig. 2). In the trials Nem19-1 and Nem17-1 eggs per g of root explained 58% of variations in root biomass, and the regression and correlation coefficients were highly significant (*p* < 0.0001) (Fig. 2). In the trials Nem18-1 and Nem16-2, eggs per g of roots were able to explained 20 and 15% of variation in root biomass, and the correlation and regression coefficients were significant (*p* < 0.001). In three trials, Nem15-1, Nem13-2, and Nem12-1 eggs per g of roots were able to explain only 7% of variation in root biomass; although, correlations and regression coefficients were significant (*p* < 0.001). In all trials 5 to 8% reductions of root biomass were estimated in each unit increase of log-egg numbers per g of root. Slopes for the regression lines were significant in all trials (*p* < 0.05 to *p* < 0.0001), and varied from −0.11 in Nem15-1 to −3.02 in Nem12-1.

## Discussion

A reliable parameter for assessing a screening trial depends on two important criteria, low variation within a trial and repeatable among trials. This study clearly indicated that nematode eggs per plant showed higher repeatability across the trials, and relatively low variations (CV) compared to other parameters. Between two other nematode resistance parameters, nematode eggs per g roots showed greater reliability and repeatability compared to visual gall rating.

Root-knot nematode eggs per g of root on a wide range of sugarcane introgression and commercial breeding lines were negatively correlated with root biomass in all but one trial and with shoot biomass in four out of the eight trials. In contrast, eggs per plant was not significantly correlated with shoot or root biomass in any trial. Visual rating of galling on plants was significantly correlated with shoot or root biomass in less than half of the trials and the correlation was not consistently negative. Sugarcane breeders evaluate large numbers of accessions each year for nematode resistance. It is important to decide which assessment parameter is appropriate and provides reliable results without delay. Resistance selection should be based on the ability of an accession line to suppress reproduction and maintain shoot and root biomass. The regression analysis indicated that nematode eggs per g of root were an indicator of varietal resistance for sugarcane to root-knot nematodes (Fig. 2).

Nematode eggs per plant has been used widely to measure the reproductive capabilities of nematodes on test plants in resistance screening trials (Bhuiyan et al., 2019; Stirling et al., 2011; Thompson et al., 2009). In our trials this parameter provided better repeatability in separating accession lines with relatively low variations in the breeding program (Table 4). As indicators for resistance, egg numbers per plant was more effective and independent of root biomass of the accessions. The negative correlations of nematode per g of root with root biomass can be useful in separating accession lines on the basis of this parameter. However, the greater variations of root biomass within an accession line, manifested in this study, rendered it less repeatable. Variation in root biomass is common among sugarcane varieties in Australia (Pierre et al., 2019).

Both the parameters eggs per plant and eggs per g root were able to separate accession lines accurately (Table 2). Two wild sugarcane accessions IJ76-333 and IJ76-370 and introgression line QBYN05-20563 showed a high level of resistance reaction to *M. javanica*. Some commercial varieties, Q249, Q241, and Q138 had intermediate level of resistance. This agrees with previous research that indicated that commercial sugarcane varieties possessed low level of resistance to *M. javanica* (Stirling et al., 2011).

Visual ratings for root-knot nematode galling were highly correlated with eggs per plant and eggs per g of root. This is in agreement with our previous work where visual ratings were correlated with extracted eggs from the sugarcane accession lines (Bhuiyan et al., 2014). Although this parameter was less reliable and repeatable compared to two other resistance parameters, visual rating has been used to screen a range of crops such as peanuts and *Psidium* species (Dong et al., 2008; Milan, 2007. Dong et al. (2007) reported the visual rating was reliable if inoculated with 8,000 or more eggs when harvest earlier, and can be used as rapid method for early selection. However, Matsuo et al. (2012) opposed the exclusive use of root galling to assess resistance, as it can cause errors in selecting for nematode resistance. They indicated that some genotypes do not produce galls in response to root-knot nematode infection even though nematode reproduction in those genotypes may be high. Due to the fibrous root system of sugarcane, it is sometimes difficult to visually assess plants for galling.

Shoot and root biomass can be used to assist in comparisons between accessions (Stirling et al., 2011). As an obligate parasite of plants, reproduction of *M. javanica* is limited by the availability of roots. If accessions with similar root biomass have different number of nematodes, then the difference is most likely due to resistance. However, if an accession has smaller root system, the lower number of nematodes could be due to the limitations in the root biomass available as a food source. In sugarcane, there is significant variation in root biomass among test accessions. In more detailed studies of advanced lines, it may be desirable to compare shoot and root biomass of inoculated and uninoculated plants of the same accessions. This would allow a direct measure of the effect of the nematode on shoot and root growth.

Greater populations of *M. javanica* in root system may not necessarily lead to a reduction of yield in some varieties. Anecdotal evidence suggested that some varieties possessed tolerance to *M. javanica*. In two glasshouse experiments comparing inoculated and uninoculated plants, the commercial variety Q208 supported the highest number of nematodes whereas it had high root and shoot biomass in both treatments (Bhuiyan et al., 2016). No information on nematode tolerant varieties is available in Australia, as field trials comparing the performance of accessions in nematode infested and non-infested soil have not been conducted. It is important to identify and select nematode tolerant accessions to improve the breeding program; therefore, more research on tolerance is warranted.

There were moderate to strong correlations for eggs per plant in accession lines among majority of the trials (Table 4), suggesting that this parameter is the most reliable indicator to assess root-knot nematode resistance in sugarcane. However, the number of eggs per g of sugarcane root was clearly related to root and shoot biomass, which may be indicative of both resistance and tolerance. Therefore, eggs per g of root may be used as a secondary selection factor for advancing germplasm in a breeding program. Further research is required to verify the resistance of sugarcane accession lines in the glasshouse and under field conditions.
